# A162 DEFINING INNATE IMMUNE CELL MECHANISMS UPON *HELICOBACTER PYLORI* MEDIATED TUMORIGENESIS

**DOI:** 10.1093/jcag/gwad061.162

**Published:** 2024-02-14

**Authors:** T Supreme, T Kim

**Affiliations:** University of Toronto, Toronto, ON, Canada; The Hospital for Sick Children, Toronto, ON, Canada

## Abstract

**Aims:**

Hypothesis: *H. pylori i*nteracts with eosinophils to inhibit degranulation, alter eosinophil gene expression and promote gastric tumorigenesis

Aim 1: Elucidate the mechanisms underlying the interactions between eosinophils and *H. pylori.*

Aim 2: Determine the role of eosinophils during *H. pylori* mediated gastric tumorigenesis.

**Methods:**

Histological and immunofluorescent staining. Hemotoxilin and eosin staining is important to analyze gastric gland structure and parietal cell loss. Immunofluorescent staining is used to detect and localize specific antigens present in cells and cell membranes.

Gastric organoids: Gastric organoids are an important tool for modelling dissease. Gastric organoids generated from adult stem cells from gastric glands depend on growth factors that mimic tissue regeneration.

Bone marrow derived eosinophils: Bone marrow progenitor cells isolated from murine femur and tibia are easily converted into maturer eosinophils with the addition of cytokine interleukin-5.

**Results:**

Addition of mouse derived eosinophils to gastric organoids significantly increases number of organoids.

To understand the relationship between eosinophils and gastric epithelial cell (GEC) growth, I designed a co-culture experiment that involved incubating mature eosinophils with gastric glands in an extracellular matrix. These findings revealed a significant increase in the number of gastric organoids per well when eosinophils were present compared to the gastric organoids without eosinophils. This observation supports the hypothesis that eosinophils may play a crucial role in promoting gastric tumorigenesis.

Exposure of mouse derived eosinophils to *H. pylori* significantly increases gastric organoid proliferation.

To explore the impact of stimulating eosinophils prior to incubation with GECs I incubated the eosinophils with *H. pylori.* As expected, there was a significant increase in the number of organoids per well with the addition of uninfected eosinophils. Interestingly, when *H. pylori* incubated eosinophils were added to the gastric glands, there was a significant increase in the number of gastric organoids per well compared to uninfected eosinophils.

**Conclusions:**

My preliminary findings have indicated that eosinophils can promote gastric epithelial stem cell proliferation. These results led me to ask how treatment of eosinophils with *H. pylori* subsequently impacts gastric organoid growth and gastric stem cell proliferation. Interestingly, I found *H. pylor*i treated eosinophils increase growth and gastric stem cell proliferation. These results support my hypothesis as increased proliferation is considered a hallmark of tumorigenesis. There little known about resident and inflammatory GI eosinophils and the role of eosinophils during tumorigenesis in unknown. A better understanding of the role eosinophils play can uncover novel mechanisms to be targeted for therapeutic intervention.

**Funding Agencies:**

CIHR

